# Prevalence and risk factors for subclinical hypothyroidism in older patients with major depressive disorder

**DOI:** 10.1186/s12877-023-04584-9

**Published:** 2024-01-04

**Authors:** Min Li, Xiu-Wen Wang, Xiao-Qian Wang, Jian-Jun Zhang, Xiang-Yang Zhang

**Affiliations:** 1 Department of Pharmaceutical and Food Engineering, Shanxi University of Chinese Medicine, Jinzhong, China; 2 Shanxi key laboratory of Chinese medicine encephalopathy, National international joint research center for molecular Chinese medicine, Shanxi University of Chinese Medicine, Shanxi Jinzhong, 030619 China; 3 Experimental Management Center, Shanxi University of Chinese Medicine, Jinzhong, China; 4 CAS Key Laboratory of Mental Health, Institute of Psychology, Chinese Academy of Sciences, 16 Lincui Rd, Beijing, 100101 China; 5 Department of Psychology, University of Chinese Academy of Sciences, Beijing, China

**Keywords:** Subclinical hypothyroidism (SCH), Major depressive disorder (MDD), Older patients, Fasting plasma glucose (FPG), Total cholesterol (TC)

## Abstract

**Background:**

Subclinical hypothyroidism (SCH) is highly correlated with major depressive disorder (MDD). However, the prevalence and risk factors for SCH in older patients with MDD have rarely been reported in China.

**Methods:**

This cross-sectional study included 266 older MDD patients with SCH was performed. Clinical and anthropometric, biochemical, and thyroid function data were collected. Depression, anxiety, and psychotic symptoms were assessed using the Hamilton Depression Scale, the Hamilton Anxiety Scale, and the Positive and Negative Syndrome Scale positive subscale, respectively.

**Results:**

Among older patients with MDD, the prevalence of SCH was 64.7% (172/266). Compared to patients without SCH, older MDD patients with SCH had a longer disease course and higher TSH, A-TG, A-TPO, HDL-C, LDL-C, TC, FPG, and systolic pressure levels (all *P* ≤ 0.002). Furthermore, disease progression (OR 1.082, 95% CI 1.020–1.147, *P* = 0.009), A-TG (OR 1.005, 95% CI 1.001–1.009, *P* = 0.017), TC (OR 2.024, 95% CI 1.213–3.377, *P* = 0.007), FPG (OR 2.916, 95% CI 1.637–5.194, *P* < 0.001), systolic pressure (OR 1.053, 95% CI 1.008–1.100, *P* = 0.022) were independently associated with SCH, in older patients with MDD.

**Conclusions:**

Our findings suggest a high prevalence of SCH in older patients with MDD. Several demographic and clinical variables were independently associated with SCH in older patients with MDD.

## Background

Major depressive disorder (MDD) is a common psychiatric disorder with a widespread problem and high prevalence, accounting for 10% of the total nonfatal disease burden [1, 2]. Epidemiologic studies showed that the lifetime prevalence of MDD in America is 16.2% [3]. In mainland China, the lifetime prevalence of MDD is 2.3% [4].

MDD is one of the most common mental health disorders among older people. The prevalence of MDD, especially among older adults, is increasing annually and is a growing concern. Men with depression represent 7–10% of the older adult population. In Portugal, the prevalence of MDD in the older population (≥ 65 years) is 7.5–12.6% [5]. Geriatric depression can increase the risk of morbidity and mortality due to physical diseases (such as cardiovascular and cerebrovascular diseases), leading to a delay in the course of the disease and a decrease in the quality of life of older adults.

The coexistence of MDD and subclinical hypothyroidism (SCH) has received increasing attention. SCH, defined as elevated thyroid-stimulating hormone (TSH) and normal free thyroxine (FT4) levels [6], is a strong risk factor for overt hypothyroidism [7, 8]. The prevalence of SCH ranged from 4 to 20% in adult population samples worldwide [9, 10], and the incidence of SCH is 8.7% in China [11]. The relationship between SCH and depression is not clear [12], but it is known that higher SCH is associated with an increased risk of MDD in older populations (≥ 50 years) [13]. Clinically, patients with MDD with and without SCH show significant differences in the tendency to present with comorbid panic disorder and a poorer response to antidepressants [14]. Furthermore, variations in thyroid function may be related to cognitive deficits associated with depression. Even patients with mild thyroid dysfunction may have cognitive deficits or psychomotor impairment [15, 16]. Furthermore, meta-analyses have shown that the risk of altered cognitive function is high in patients aged < 75 years with SCH [17]. SCH is also associated with treatment-resistant depression (TRD). Patients with TRD have higher serum TSH levels and higher rates of SCH [18, 19]. Thus, failure to diagnose SCH may contribute to delayed treatment response for depression. Several studies have shown an association between SCH and current depressive symptoms and a lifetime history of MDD. However, the relationship between depressive symptoms and SCH remains controversial, particularly in older patients with MDD. A meta-analysis showed that SCH was associated with depression in younger patients (< 60 years) but not in older patients (≥ 60 years) [20]. Another study showed that the prevalence of both depression and hypothyroidism is high in older adults and that SCH increases the risk of depression [21]. Another recent review found no relationship between thyroid hormone levels and MDD [22]. These inconsistent findings may be attributed to the heterogeneity of the study populations, differences in study design, or sex differences. SCH also exhibits a sex-differentiated pathology. Studies have shown that SCH may affect up to 22% of older women (> 60 years) and is slightly less prevalent in older men [10].

Studies have shown an association between thyroid dysfunction and lipids or depression [23, 24]. Thyroid dysfunction plays a critical role in metabolic regulation, and SCH has been associated with metabolic parameters such as FBG, lipid metabolism, blood pressure levels, and BMI [25]. SCH is associated with elevated levels of TC and LDL-C levels, especially in women and those older than 60 years [26, 27]. SCH is also associated with diastolic hypertension, reduced vasodilation due to impaired endothelial function, increased arterial stiffness, and impaired left ventricular systolic and diastolic function at rest and during exercise [28]. This evidence suggests the need for physiological measurements in older patients with MDD to investigate the risk factors associated with SCH.

Studies on the prevalence of SCH in older patients with depression are scarce. We recruited 266 first-episode and drug-naive patients with MDD aged ≥ 50 years in mainland China as study subjects and collected clinical data and lipid metabolism and neuroendocrine-related biomarkers. The main objectives of this study were (1) to investigate the prevalence of SCH in older patients with MDD and (2) to investigate the risk factors associated with SCH in older patients with MDD.

## Methods

### Study design and subjects

We recruited patients from the psychiatric clinic of the First Affiliated Hospital of Shanxi Medical University in China from 2016 to 2017. This cross-sectional study was approved by the Institutional Review Board of Shanxi Medical University. Before enrollment, all participants were informed about the study and signed an informed consent form.

A total of 266 participants were recruited for the study. Two psychiatrists independently confirmed the diagnosis of MDD using the Chinese version of the Structured Clinical Interview for DSM-IV. All patients met the following inclusion criteria: (1) age ≥ 50 years, Han Chinese ancestry; (2) met criteria diagnosis of MDD; (3) first episode and drug naive (FEDN) patients; and (4) total score on the 17-item Hamilton Rating Scale for Depression (HAMD-17) ≥ 24. Exclusion criteria were: (1) substance use disorder, (2) major physical illness, (3) personality disorder, and (4) pregnancy or lactation. Sociodemographic information, clinical measurements, and biochemical parameters (including thyroid function assessment) were collected sequentially.

### Collection of sociodemographic information

We used a standard questionnaire to collect sociodemographic information, including age, sex, marital status, years of education, age of onset, duration of illness, and suicide attempts.

### Clinical measurement

Prior to the study, two qualified psychiatrists were trained in the use of the following three scales: After training, the correlation coefficients between the variables were greater than 0.8.

The HAMD-17, Hamilton Anxiety Scale (HAMA-14), and Positive and Negative Syndrome Scale (PANSS) subscales were used to assess depression, anxiety, and psychotic symptoms, respectively. Based on previous studies, we used a cutoff score of 24 to select patients with MDD [29, 30], 18 to divide patients into groups with or without anxiety symptoms [31], and 15 to divide patients into groups with or without psychotic symptoms. We used the CGI-S scale from 1 (normal) to 7 (most severe) to assess the overall severity of illness [32].

We collected information on suicide attempts through interviews. Each participant was asked the same question: “Have you attempted suicide in your lifetime?” If the answer was “yes,” the participant was defined as having attempted suicide, and we asked for the following details: number of suicide attempts, date of each suicide attempt, and exact method.

### Biochemical parameter measurements

Peripheral venous blood was collected between 07:00 and 09:00 a.m. after an overnight fast. Samples were tested before 11 a.m. on the same day that the clinical data were collected for testing. Hospital laboratory technicians used an automated biochemistry analyzer to measure blood glucose levels and lipid profiles, including triglyceride (TG), total cholesterol (TC), and high- and low- density lipoprotein cholesterol (HDL-C and LDL-C) levels.

A Roche C6000 Electrochemiluminescence Immunoassay Analyzer (Roche Diagnostics, Indianapolis, IN, USA) was used to detect TSH, FT4, thyroid peroxidase antibody (A-TPO), thyroglobulin antibody (A-TG), and free triiodothyronine (FT3). The normal range was 0.27–4.20 mIU/L for TSH, 3.10–6.8 pmol/L for FT3, 10–23 pmol/L for FT, 4.0–34 IU/L for TPOAb, and 0–115 IU/L for TGAb [33]. SCH was defined as elevated TSH (> 4.2 mIU/L) and normal FT4 [34, 35].

### Statistical analysis

First, the normality of all variables was tested using the Shapiro–Wilk test and the homogeneity of variance was determined using the Levene test. The X^2^ test was used to compare categorical variables, which were expressed as frequencies and percentages. All normally distributed continuous variables were expressed as mean ± SD, and non-normally distributed variables were expressed as median (interquartile range). Comparisons of continuous variables conforming to a normal distribution were performed using analysis of variance (ANOVA), and non-normally distributed variables were tested using the Kruskal–Wallis rank-sum test. To further explore the effect of each clinical factor on older MDD patients without SCH or with SCH, a multivariate unordered binary logistic regression model was constructed to clarify the effect of each factor on SCH levels (without SCH or with SCH). The TSH level was considered the dependent variable. All statistical analyses were performed using SPSS 18.0, and all *p*-values were calculated using two-tailed tests, with significance set at *P* < 0.05.

## Results

### Differences in physical and biochemical parameters between older MDD patients with and without SCH

Demographic and clinical characteristics of all participants are shown in Table 1. Among the 266 older patients with MDD (age ≥ 50, 68 men and 198 women), the prevalence of SCH was 64.7% (172/266). The Kolmogorov–Smirnov rank test showed that the TSH, FT3, TC, and LDL-C levels were normally distributed. The following variables were not normally distributed: age, age at onset, disease course, years of education, HAMA, HAMD, positive total score, A-TG, A-TPO, FT4, TG, FPG, HDL-C, BMI, systolic pressure, and diastolic pressure (all *P* < 0.05). Among the 266 older MDD patients, compared to older MDD patients without SCH, patients with SCH have a longer disease progression (*P* < 0.001), longer married (*P* = 0.007), higher HAMD values (*P* < 0.001), higher positive total score values (*P* = 0.002), higher CGI (*P* = 0.004), higher BMI (*P* = 0.008), higher attempted suicide (*P* = 0.017), and higher levels of TSH (*P* < 0.001), A-TG (*P* = 0.002), A-TPO (*P* < 0.001), HDL-C (*P* < 0.001), LDL-C (*P* < 0.001), TC (*P* < 0.001), FPG (*P* < 0.001), systolic pressure (*P* < 0.001). After Bonferroni correction, the disease course, HAMD, TSH, positive total score, A-TG, A-TPO, HDL-C, LDL-C, TC, FPG, and systolic pressure remained significant (*P* ≤ 0.002).

**Table 1:** Demographics and clinical characteristics of patients with and without SCH in older patients with MDD

	Total	Patients without SCH (0.27 ≤ TSH ≤ 4.2)	Patients with SCH (TSH > 4.2)	*F* or χ2	*P* value
**Older MDD Patients (%)**	266	94 (35.3%)	172 (64.7%)		
**Gender**				0.336	0.562
male, N (%)	68(25.6%)	26(27.7%)	42(24.4%)		
female, N (%)	198(74.4%)	68(72.3%)	130(75.6%)		
**Age (years)**	54(51, 57)	54(51, 57.25)	54(51, 56)	0.516	0.473
**Age of onset (years)**	54(51, 56)	54(51, 57.25)	53.5(51, 56)	1.467	0.226
**Disease course (month)**	6(4, 12)	4(2.375, 8.25)	8(5, 13)	24.720	0.000**
**Years of education (years)**	9(9, 12)	9(9, 12)	9(9, 12)	0.542	0.462
**Marital status**				7.342	0.007*
Married	257(96.6%)	87(92.6%)	170(98.8%)		
Unmarried	9(3.4%)	7(7.4%)	2(1.2%)		
**HAMD**	31(29, 32)	29(27, 31)	32(29, 33)	34.204	0.000**
**HAMA**	21(18.75, 23)	21(18, 23)	21(19, 23)	1.988	0.159
**Positive total score**	7(7, 9)	7(7, 7)	7(7, 10)	9.183	0.002*
**TSH**	5.399 ± 2.721	2.576 ± 1.084	6.942 ± 2.015	380.660	0.000**
**A-TG**	22.19 (14.313, 59.689)	20.175 (13.718, 27.708)	23.305(15.285, 115.393)	10.035	0.002*
**A-TPO**	17.67 (11.995, 39.115)	14.885(10.035, 26.635)	22.165(12.51, 56.808)	13.066	0.000**
**FT3**	4.829 ± 0.750	4.766 ± 0.672	4.863 ± 0.789	1.030	0.311
**FT4**	16.23(14.29, 18.658)	16.86(14.41, 19.975)	16.07(14.29, 18.288)	3.309	0.069
**HDL-C**	1.19(0.98, 1.3925)	1.265(1.11, 1.493)	1.13(0.91, 1.3)	17.280	0.000**
**LDL-C**	3.062 ± 0.830	2.604 ± 0.624	3.313 ± 0.822	85.555	0.000**
**TC**	5.390 ± 1.074	4.672 ± 0.807	5.782 ± 0.998	53.104	0.000**
**TG**	1.99(1.45, 2.76)	1.81(1.47, 2.458)	2.155(1.42, 2.885)	4.936	0.026
**FPG**	5.4(4.9575, 5.87)	5.07(4.76, 5.473)	5.57(5.143, 5.98)	32.222	0.000**
**BMI**	24.19 (23.2, 25.48)	23.7(22.978, 25.358)	24.375(23.443, 26.021)	6.949	0.008*
**Systolic pressure**	128(122, 134)	126(119.75, 130)	130(124, 135)	14.642	0.000**
**Diastolic pressure**	78(72, 85)	78(70, 85.25)	78(72, 85)	0.334	0.563
**CGI**				11.293	0.004*
5	80(30.1%)	35(37.2%)	45(26.2%)		
6	104(39.1%)	42(44.7%)	62(36.0%)		
7	82(30.8%)	17(18.1%)	65(37.8%)		
**Severe anxiety**				1.240	0.266
NO	223(83.8%)	82(87.2%)	141(82.0%)		
YES	43(16.2%)	12(12.8%)	31(18.0%)		
**Attempted suicide**				5.660	0.017*
NO	201(75.6%)	79(84%)	122(70.9%)		
YES	65(24.4%)	15(16%)	50(29.1%)		
**Psychotic symptoms**				2.901	0.089
NO	224(84.2%)	84(89.4%)	140(81.4%)		
YES	42(15.8%)	10(10.6%)	32(18.6%)		

Note: Not SCH: 0.27 mIU/L ≤ TSH ≤ 4.2 mIU/L; SCH: TSH > 4.2 mIU/L and norma FT4; *P < 0.05; ** *P* < 0.001

### Clinical correlates of SCH in older patients with MDD

Binary logistic regression analysis was performed to analyze the risk factors for SCH in older patients with MDD. We selected SCH as the dependent variable and variables that differed significantly in the univariate analysis as independent variables. As shown in Table 2, FPG (B = 1.070, *P* < 0.001, OR: 2.916, 95% C.I.: 1.637–5.194), TC (B = 0.705, *P* = 0.007, OR: 2.024, 95% C.I.: 1.213–3.377), disease course (B = 0.079, *P* = 0.009, OR: 1.082, 95% C.I.: 1.020–1.147), A-TG (B = 0.005, *P* = 0.017, OR: 1.005, 95% C.I.: 1.001–1.009), and systolic pressure (B = 0.051, *P* = 0.022, OR: 1.053, 95% C.I.: 1.008–1.100) were independently associated with SCH in older patients with MDD.

**Table 2:** Results of the binary logistic regression model

	B	S.E.	Wald	df	*P* Value	OR	95%C.I.	
							Lower Limit	Upper Limit
**Constant**	-18.916	3.882	23.744	1	0.000**	0.000		
**FPG**	1.070	0.295	13.194	1	0.000**	2.916	1.637	5.194
**TC**	0.705	0.261	7.288	1	0.007*	2.024	1.213	3.377
**Disease Course**	0.079	0.030	6.822	1	0.009*	1.082	1.020	1.147
**A-TG**	0.005	0.002	5.697	1	0.017*	1.005	1.001	1.009
**Systolic Pressure**	0.051	0.022	5.281	1	0.022*	1.053	1.008	1.100

Note: *P < 0.05;** *P* < 0.001

## Discussion

To the best of our knowledge, this is the first clinical study to show that the prevalence of SCH in older Chinese patients with MDD was 64.7%. FPG, TC, disease course, A-TG, and systolic pressure are risk factors for SCH in older patients with MDD.

We found a high prevalence of SCH (64.7%) in older patients with MDD, which is consistent with the results of previous studies [33, 36]. Lang et al. showed that the prevalence of SCH was 60.7% in patients aged 18–60 years, similar to our study’s findings.

Aging is also associated with an increased incidence of hypothyroidism. A survey on the prevalence of thyroid diseases in 10 cities in China showed that the prevalence of SCH in older adults (65 years) was 19.87% [37]. An epidemiologic survey of 78,470 adults in 31 provinces showed that the prevalence of SCH was 16.13% in people aged 60–69 years and 19.09% in people aged ≥ 70 years old [38]. In our study, women with MDD were found to be more susceptible to SCH (Table 1). Previous studies have also shown that SCH affects women more than men [20, 39, 40]. Sex differences also exist in the incidence of thyroid and neurological disorders [41]. TSH may also be one of the key signaling molecules that regulate different brain signals in a male- and female-specific manner. The reasons for these differences are complex and may include differences in brain structure, function, and stress response, as well as differences in reproductive hormone exposure, social expectations, and experience [42].

Our study found that FPG, TC, disease progression, A-TG, and systolic pressure were risk factors for SCH in older patients with MDD, whereas a previous study with a larger sample (n = 1706) showed that suicide attempts and psychiatric symptoms were associated with severe SCH [33]. These different findings may be due to the different age groups and sample sizes of the study populations. We studied patients older than 50 years, and Lang et al. studied patients aged 18–60 years with SCH from MDD [33].

Our study found that FPG level was a risk factor for SCH in older patients with MDD. Patients with MDD exhibit abnormal glucose metabolism, including elevated fasting blood glucose, insulin, and glucagon [43]. The relative risk of diabetes in patients with MDD is 1.2–2.6 times than that in non-depressed patients [44]. Liu et al. showed that severe anxiety, PANSS score, and suicidal behavior were positively associated with blood glucose levels in Chinese patients with MDD and that glucose metabolism disorders may accelerate the development of MDD [45]. Peng et al. also found a significant association between elevated FPG levels and MDD, and a higher prevalence of MDD was observed in older adult women [46]. Both the thyroid and pancreatic islets are endocrine organs, and there is a hormonal balance between them. Pancreatic islets are organs that break down and produce blood sugar. Hypothyroidism can affect blood glucose metabolism by lowering glucose metabolism and blood glucose levels. Previous studies have also shown that hypothyroidism is associated with insulin resistance [47–49]. Impaired insulin sensitivity is typically attributed to decreased intracellular glucose utilization and decreased glucose transporter (GLUT4) translocation. Reduced glycogen synthesis and oxidation may also contribute to this condition [30]. As thyroid hormones regulate hepatic glycogenesis, lipogenesis, and lipolysis, they play an important role in carbohydrate metabolism. Thyroid hormones regulate the expression of glucose transporters, adenosine monophosphate-activated protein kinase, and acetyl-CoA carboxylase in skeletal muscle [28]. Therefore, clinicians should pay attention to the connection between this aspect in the daily diagnosis and treatment process, early prevention and treatment, and reduce the risk of thyroid disease in patients.

Our study also found that the TC level was a risk factor for SCH in older patients with MDD. Thyroid hormones are involved in all aspects of lipid metabolism, including lipid synthesis, mobilization, and degradation. SCH affects the metabolic rate of cholesterol, leading to an increase in total cholesterol levels. The role of thyroxine in human blood lipid metabolism is to promote the synthesis, increase cholesterol, and promote the excretion of cholesterol and bile metabolites. When thyroxine is reduced or insufficient, although the synthesis of cholesterol is also reduced, the rate of excretion is more obvious, the degree of which is significantly greater than the rate of cholesterol synthesis; therefore, the concentration of cholesterol in the blood increases. Thyroid hormones have also been suggested to have a major influence on lipid degradation. One possible explanation is that thyroxin is required for LDL receptor gene expression and synthesis [50]. Moreover, SCH may affect cholesterol excretion, lipoprotein B, and the activities of hepatic lipase and cholesteryl ester transport proteins [23, 51].

We found that A-TG level was a risk factor for SCH in older patients with MDD. Serum A-TG is a specific indicator for the diagnosis of autoimmune thyroid diseases, and hypothyroidism is closely associated with A-TG [52]. In women, the rate of positive A-TG detection increases with age, up to 18% in those over 40 years, which may be an early response to autoimmune thyroid disease [53]. Therefore, clinical attention should be paid to the detection of A-TG indicators for the diagnosis of older MDD patients with SCH.

In addition, we found that disease progression and systolic pressure were risk factors for SCH in older patients with MDD. To the best of our knowledge, this is the first study to show that these two factors are risk factors for SCH. Compared to MDD patients without SCH, older patients with SCH have a longer disease course and higher systolic pressure. We speculate that the longer the disease course of older patients with MDD, the longer the duration of loss of appetite, which may lead to insufficient iodine intake. Iodine nutritional status is also an important factor in the prevalence of thyroid dysfunction in older adults [38]. Thus, inadequate iodine intake can lead to decreased thyroid hormone production, which can cause hypothyroidism. If patients with hypothyroidism are left untreated for a long period of time, thyroid hormone levels will decrease and metabolism will slow down. Most patients have elevated blood lipid levels, increased arteriosclerosis, and poor vascular elasticity, all of which can lead to elevated blood pressure. The underlying pathophysiological mechanisms of hypertension in hypothyroidism may involve changes in circulating catecholamines, catecholamine receptors, and the renin-angiotensin-aldosterone system [50]. One study found that 30% of patients with SCH had a systolic blood pressure > 140 mmHg, compared with 16.7% in the normal thyroid function group [54]. Therefore, clinical attention should be paid to early diagnosis and treatment of this condition.

The incidence of SCH and MDD in the general population is increasing annually. Analysis of the prevalence and risk factors of subclinical hypothyroidism in older patients with MDD also provides a reference for public health services. Regular medical examinations of high-risk populations should focus on monitoring FPG, TC, disease progression, A-TG levels, and systolic pressure. In addition, all sectors of the community should strengthen scientific education and publicity on SCH and improve the understanding of SCH among older patients with MDD to guide its prevention and control.

This study had several limitations. First, current international definitions of older adults are not consistent. For the age definition of older adults, we used an age range that was more appropriate for the Chinese population, which may have led to results inconsistent with other studies. Second, in this study, we were unable to obtain TSH levels before the onset of MDD and instead recommended that patients undergo repeated thyroid hormone tests at least three months apart to clarify the diagnosis. Therefore, the results of our study are limited to future longitudinal cohort studies to better elucidate the relationship between the prevalence of SCH and the duration of depression. Third, because most of our samples were sent to the hospital laboratory for routine testing on a case-by-case basis, many batches of samples were used and some assay parameters (coefficient of variation and limit of detection) were not collected, which may have contributed to bias in laboratory measurements. We did not detect the levels of novel biomarkers, for example miR-139-5p, which has recently been proposed as a biomarker in the diagnosis of MDD [55, 56]. Fourth, there was no healthy control group, and all patients with MDD were from the outpatient department of a general hospital in China. Therefore, our findings are limited and cannot be generalized to other settings, which needs to be confirmed in future studies.

## Conclusion

In conclusion, our study showed, for the first time, that the prevalence of SCH in older Chinese patients with MDD was 64.7%. Our study also found that FPG, TC, disease course, A-TG levels, and systolic pressure were risk factors for SCH in older patients with MDD. Therefore, FPG, TC, disease course, A-TG, and systolic pressure testing in older patients with MDD, which may be predictive of SCH, should be strengthened in clinical practice.

## Data Availability

The data that support the findings of this study are available from the corresponding author upon reasonable request.
